# A combined effort to avoid strongyle infection in horses in an oceanic climate region: rotational grazing and parasiticidal fungi

**DOI:** 10.1186/s13071-018-2827-3

**Published:** 2018-04-12

**Authors:** José Ángel Hernández, Rita Sánchez-Andrade, Cristiana Filipa Cazapal-Monteiro, Fabián Leonardo Arroyo, Jaime Manuel Sanchís, Adolfo Paz-Silva, María Sol Arias

**Affiliations:** 1 Equine Diseases Study Group (COPAR, GI-2120), Animal Pathology Department, Veterinary Faculty, Santiago de Compostela University, 27002 Lugo, Spain; 2 Parasitología, Universidad de la República (Regional Norte), Salto, Uruguay

**Keywords:** Horses, Strongyles, Rotational pasturing, Pelleted feed, Integrated control, *Duddingtonia flagrans*, *Mucor circinelloides*

## Abstract

**Background:**

An approach to preventing strongyle infection in horses was tested, comprising rotational pasturing and the administration of spores of two parasiticidal fungi, *Mucor circinelloides* and *Duddingtonia flagrans*.

**Methods:**

Twenty-two adult Spanish Sport Horses were dewormed with ivermectin (1 mg *pour-on*/kg body weight) and then randomly divided into three groups. G-1 was maintained with continuous grazing, and G-2 and G-3 were kept on a four-paddock rotation system. Commercial pelleted feed (2.5 kg/horse) was supplied to G-1 and G-2 twice a week; horses in G-3 received pellets containing 2 × 10^6^ spores/kg of each fungus. Fecal samples were analyzed by the flotation method to estimate the reduction in the fecal egg counts (FECR), the percentage of horses shedding eggs (PHR), and the egg reappearance period (ERP).

**Results:**

Third-stage larvae were identified in fecal pats as *Cyathostomum* (*sensu lato*) types A, C and D, *Gyalocephalus capitatus*, *Triodontophorus serratus*, *Poteriosthomum* spp., *Strongylus vulgaris* and *S. edentatus*. Two weeks after treatment, the FECR values were 100% in G-1, 96% in G-2 and 99% in G-3; the PHR values were 100% in G-1, 75% in G-2 and 88% in G-3. A strongyle ERP of 6 weeks was observed in G-1, ERP of 10 weeks was observed in G-2, and ERP of 16 weeks was observed in G-3. The counts of eggs per gram of feces (EPG) were > 300 EPG in G-1 and G-2 but remained below 250 EPG in G-3 throughout the observation period of 12 months.

**Conclusions:**

These results suggest that horse strongyle infection could be decreased by combining rotational pasturing with feeding pellets containing the spores of parasiticidal fungi.

## Background

Horses maintained on pasture receive important benefits that include not only nutritional benefits but also the opportunity to exercise and to socialize [1]. Many helminths develop an external phase of their life-cycle in the soil, including trematodes, cestodes and nematodes [2]; thus horses on pasture are at risk of infection by consuming the infective stages of these helminths with herbage [3]. Among the gastrointestinal nematodes, strongyles are commonly reported in grazing horses. Eggs passed in feces develop a larva 1 (L1) stage inside, which hatches and molts into L2 and then L3, the infective stage [4].

High numbers of viable L3s have been recorded as surviving for long periods in pastures in oceanic climate areas, and even in regions where cold climate conditions occur during certain months of the year [5, 6]. An oceanic climate, also termed a maritime or marine west coast climate, is characterized by temperatures within a narrow range and reliable rainfall throughout the year, with warm summers and mild, cool winters. This climate can be found in regions along the west coasts at the middle latitudes of all continents, and in Chile, New Zealand and Tasmania and is responsible for grass that grows almost continuously, except in winter [7].

With the aim of removing parasitic infective stages from the environment where horses feed, grassland rotation is recommended [8]. Grassland rotation is a practice wherein animals are moved among different pastures, with each pasture having two phases, grazing and resting. Some strategies of biological control against strongyles include the utilization of soil saprophytic fungi such as *Duddingtonia flagrans* or *Monacrosporium thaumassium* [9], which are able to create traps in their mycelium where larvae are captured, immobilized and finally digested [10]. Therefore, administration of those spores has been advised as an appropriate procedure to prevent horse infection by strongyles [11, 12]. As occurs with other ovicidal fungi, *Mucor circinelloides* can attach to the egg-shell of the parasite, penetrate inside and destroy the inner content [13, 14]. An antagonistic effect of *M. circinelloides* has been shown on the eggs of helminths such as *Fasciola hepatica* and *Ascaris suum* that are shed in feces of cattle and pigs, respectively [15]. Recently, the manufacturing of pelleted feed with spores of *M. circinelloides* and *D. flagrans* has been suggested as an easy way to ensure the presence of fungal stages in the feces, where strongyle eggs hatch and develop to larvae [14, 16]. Herein, the possibility of enhancing the beneficial effect of rotational grazing by providing pelleted feed manufactured with the spores of *M. circinelloides* and *D. flagrans* has been evaluated.

## Methods

### Soil saprophytic fungi

Spores of *Mucor circinelloides* (ovicidal activity) and *D. flagrans* (larvicidal activity) were cultured in the submerged medium COPFr (patent Nr PCT/ES2014/070110) for 1.5–2 months at room temperature, until a concentration ≥ 1 × 10^8^ spores/l medium was reached [17]. These fungal species were isolated in the Laboratory of the COPAR Research Group (University of Santiago de Compostela, Spain) [18].

The spores were added during the industrial manufacturing (mixing phase) of commercial pelleted feed (ProHorse Club®, Nanta, Spain), at a concentration of 2 × 10^6^ spores/kg concentrate of each fungus [16, 19].

### Horse management

This study was carried out in a stud farm for Spanish Sport Horses (SSHs) located in Xul (Friol, Lugo, North-West (NW) Spain, 42°58'49"N, 7°48'45"W), which contains 22 adult SSHs. Six of the horses are kept stabled and are exercised twice a day by jumping, then graze for 1–2 h in the same paddock (approximately 4.5 ha) every day (continuous grazing) before returning to their stalls.

The other 16 SSHs are maintained outdoors and are dedicated to recreation (riding along the forest). There are eight fenced wooded meadows, each approximately 2.5 ha, that are available on the stud farm, and two groups of pastures are considered to enable horse management; eight of the SSHs feed on four meadows (named A-D) throughout the year, and the other eight horses pasture on the other four meadows (called 1–4). In this way, a four-paddock rotation consisting of six weeks grazing and 18 weeks rest for each pasture is conducted observed. Every 1.5 months, horses are moved to the next pasture; thus, the rotation was completed after six months. Paddocks are randomly decided each year, and all horses graze all the pastures after two years.

Waterers are placed in each meadow to provide water *ad libitum*, and feeders are supplied where pelleted feed is provided twice a week; wheat straw and barley are provided when grass is scarce (from December to February). No actions have been previously performed on the pastures to reduce the presence of parasites.

### Climatic parameters

Data regarding the temperature (maximum, minimum and average ), relative humidity (%), frost days, rainfall (l/m^2^) and water balance (l/m^2^) were collected monthly from an automatic weather station (Corno do Boi, Friol, Lugo, NW Spain, 43°02'24"N, 7°53'24"W) located 5 km away from the farm and at the same altitude.

### Experimental design

The experiment was carried out between September 2014 and October 2015. Based on previous assays involving selective therapy [20], a threshold of 300 strongyle eggs per gram of feces (EPG) was established as the criterion for horse deworming at the beginning of the study. The horses received ivermectin *pour-on* [1 mg of Noromectin 0.5%/kg body weight (bw), (Norbrook Laboratories, Newry, UK)] [7, 20] and were classified into three groups:

(i) G-1: six SSHs maintained under a continuous grazing regimen and each receiving a total of 2.5 kg of pelleted feed twice weekly (on Monday and Thursday);
(ii) G-2: eight SSHs maintained under a rotational grazing regimen and each provided 2.5 kg of pellets twice weekly;
(iii) G-3: eight SSHs kept under a rotational grazing regimen and each provided 2.5 kg of pellets containing spores of *M. circinelloides* and *D. flagrans* twice weekly.

Horses remained in the same group throughout the entire trial.

### Coprological probes

Over the course of one year, feces were collected individually from the rectums of the horses. Five grams of each sample were analyzed by using the flotation test and saturated NaCl solution (ρ = 1.20 g/ml; sensitivity = 30 eggs/gram, EPG) [20].

The strongyle species infecting the horses was identified through the analysis of fecal cultures. Prior to deworming, ten grams of feces were taken from each horse and pooled according to their group. Then, the pats were incubated for 19 days at 22–25 °C. After being collected by using the Baermann technique, third-stage larvae (L3) were identified under an optical microscope (Leica DM2500; Leica Microsistemas, Barcelona, Spain) in accordance with morphological keys [21].

The presence of spores of *M. circinelloides* and *D. flagrans* in the feces of the horses in G-3 was also investigated. During the first five days of feeding the pellets with fungal spores, five grams of feces were individually taken from each horse in this group, emulsified in 40 ml water and passed through a 150 μm sieve. Two 15 ml/tubes were filled with the filtered solution and centrifuged at 1500× *rpm* for 5 min. After discarding the supernatant, the sediment was re-suspended in 2 ml water. Aliquots of 50 μl were placed between a glass slide and a coverslip, and observed under an optical microscope for spore identification (10–20×).

### Evaluation of the efficacy

The efficacy of the strategy against the strongyles was assessed by calculating the values of the FECR (fecal egg-count reduction) and the PHR (reduction of the number of horses positive to the flotation test) [22]:

$$ \mathrm{FECR}\ \left(\%\right)=\left[1\hbox{--} \left({\mathrm{FEC}}_{\mathrm{post}\hbox{-} \mathrm{treatment}}/{\mathrm{FEC}}_{\mathrm{pre}\hbox{-} \mathrm{treatment}}\right)\right]\times 100 $$

$$ \mathrm{PHR}\ \left(\%\right)=\left[1\hbox{--} \left(\mathrm{No}.\mathrm{of}\ {\mathrm{positive}\ \mathrm{horses}}_{\mathrm{post}\hbox{-} \mathrm{treatment}}/\mathrm{No}.\mathrm{of}\ {\mathrm{positive}\ \mathrm{horses}}_{\mathrm{pre}\hbox{-} \mathrm{treatment}}\right)\right]\times 100 $$

The egg reappearance period (ERP) was estimated by considering the week after treatment when the FECR values dropped below 90% [22].

### Adverse effects

The analysis of possible side effects in the G-3 horses after the ingestion of pellets with spores was performed by checking the activity of the digestive, reproductive and respiratory systems, and the skin integrity, in search of any disorder [16]. The possibility of pellets having strange odors or consistencies, or fungal growth, was checked.

### Statistical analysis

The FECR and PHR values are expressed as percentages and 95% confidence intervals. The egg-output kinetics are represented as the mean ± 2SD. Because the Kolmogorov-Smirnov test showed that the values of strongyle egg-output were not normally distributed (statistic = 0.088, *P* = 0.001), these data were analyzed by means of the non-parametric Kruskal-Wallis and Mann-Whitney U two-sided tests (α = 0.05) [24]. Significant differences were considered when *P* < 0.05, and the time points with significant differences between groups are indicated in the figures. All tests were done using SPSS for Windows (v. 20.0; SPSS Inc., Chicago, IL, USA).

## Results

### Climatic variations

**Fig. 1:**
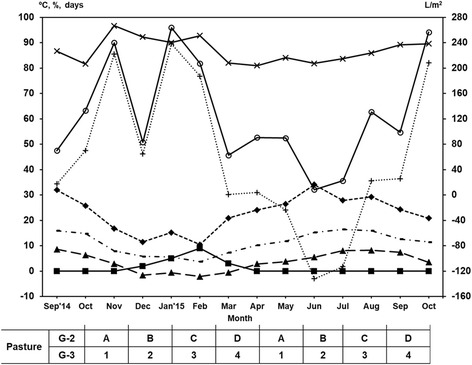
Variations of climatic parameters in an oceanic climate area (NW Spain). *Key*: ♦, maximum temperature (°C); ▁, average temperature (°C); ▲, minimum temperature (°C); ✕, relative humidity (%); ■, number of frost days; ○, rainfall (l/m^2^); ┼, water balance (l/m^2^)

As summarized in Fig. 1, temperatures oscillated between 34 °C (June) and -2 °C (February), and frost days were recorded from December to March. Rainfall peaked in January, and the lowest values were achieved in June; relative humidity higher than 81% was observed throughout the study. The water balance, or the difference between the accumulated rainfall and evapotranspiration, was negative from May to July.

### Strongyle species identified

Eggs of strongyles were observed in the feces of all horses. In the fecal pats, the larvae were identified as *Cyathostomum* (*sensu lato*) type A (63%), type C (14%) and type D (15%); *Gyalocephalus capitatus* (1%), *Triodontophorus serratus* (2%), *Poteriosthomum* spp. (2%), *Strongylus vulgaris* (2%) and *S. edentatus* (1%).

### Fungal spores in the feces

**Fig. 2:**
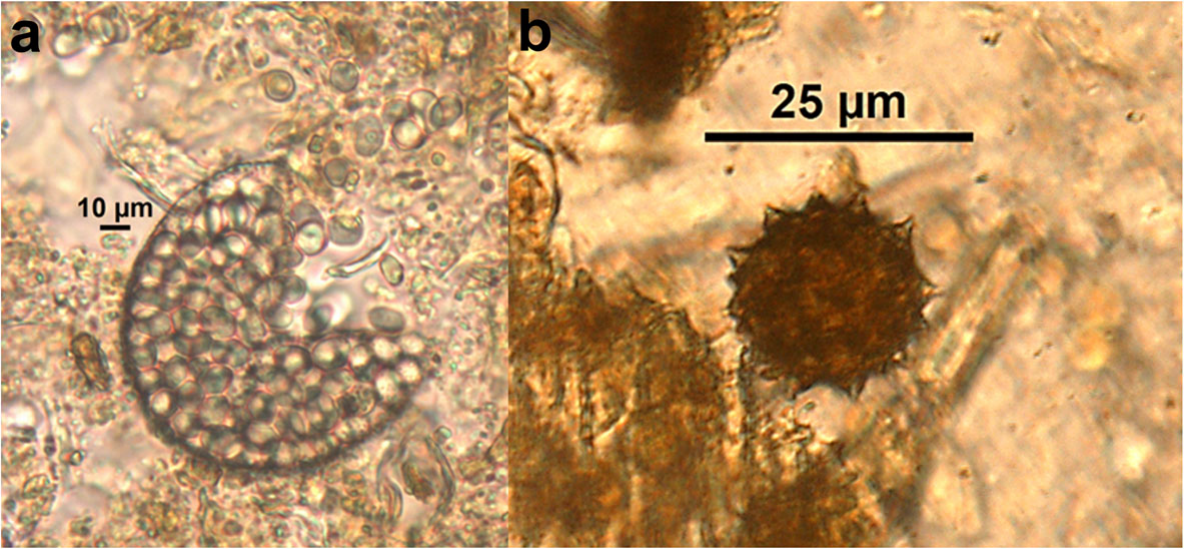
Spores of *Mucor circinelloides* (**a**) and *Duddingtonia flagrans* (**b**) in feces of Spanish Sport Horses under a four-paddock rotation system, two days after provided pellets containing 2 × 10^6^ spores/kg of each fungus

Spores of *M. circinelloides* and *D. flagrans* were first detected in all the G-3 horses two days after pellets were provided (Fig. 2).

### Efficacy of deworming

At the beginning of the study, values of strongyle egg-output higher than 300 EPG were observed in all the horses. As summarized in Table 1, the fecal counts of strongyle eggs were reduced in the three groups within two weeks after the administration of the macrocyclic lactone; the FECR values were 100% in G-1 horses, 96% in G-2 horses and 99% in G-3 horses. A PHR value of 100% was achieved in G-1, 75% in G-2 and 88% in G-3.

**Table 1:** Values of fecal egg count reduction (FECR) and coprological positive horses reduction (PHR) in Spanish Sport Horses under different pasturing regimes in an oceanic climate area (NW Spain)

WAT	Continuous grazing		Rotational grazing			Rotational grazing + parasiticide fungi		
	G-1 (*n* = 6)^a^		G-2 (*n* = 8)^b^			G-3 (*n* = 8)^c^		
	FECR (95% CI)	PHR (95% CI)	Pasture	FECR (95% CI)	PHR (95% CI)	Pasture	FECR (95% CI)	PHR (95% CI)
2	100	100	A	96 (94–98)	75 (50–100)	1	99 (98–100)	88 (65–100)
3	99 (97–99)	83 (46–100)		96 (94–98)	75 (50–100)		98 (97–99)	88 (65–100)
4	91 (87–93)	50 (10–90)		93 (91–96)	63 (29–96)		98 (96–99)	75 (45–100)
5	90 (87–93 )	50 (10–90)		92 (90–95)	63 (29–96)		96 (95–98)	75 (45–100)
6	80 (76–84)	0		92 (89–95)	50 (15–85)		96 (94–98)	75 (45–100)
7	72 (68–77)	0	B	92 (89–95)	50 (15–85)	2	95 (94–97)	75 (45–100)
8	54 (49–59)	0		90 (87–93)	38 (4–71)		95 (93–97)	63 (29–96)
9	52 (47–57)	0		91 (88–93)	38 (4–71)		94 (93–96)	63 (29–96)
10	51 (46–56)	0		77 (72–81)	38 (4–71)		93 (9195)	63 (29–96)
12	17 (13–20)	0		69 (65–74)	38 (4–71)		93 (9195)	50 (15–85)
14	15 (12–19)	0	C	58 (53–63)	0	3	91 (8893)	50 (15–85)
16	14 (11–18)	0		39 (34–44)	0		83 (79–86)	38 (4–71)
20	3 (1–4)	0	D	24 (20–28)	0	4	74 (70–77)	38 (4–71)
24	0	0		22 (18–26)	0		63 (59–67)	25 (0–55)
26	0	0	A	0	0	1	60 (56–65)	0
30	0	0		0	0		60 (56–65)	0
32	0	0	B	0	0	2	64 (60–68)	0
36	0	0		0	0		61 (57–66)	0
38	0	0	C	0	0	3	54 (50–59)	0
42	0	0		0	0		60 (56–65)	0
44	0	0	D	0	0	4	59 (55–63)	0
48	0	0		0	0		54 (50–58)	0

*Abbreviations*: *WAT* weeks after treatment, *CI* confidence interval, *FECR* fecal egg count reduction, *PHR* positive horses reduction

^a^G-1: horses dewormed [1 mg Ivermectin/kg bw *pour on*, Noromectin 0.5%, (Norbrook Laboratories, Newry, UK)], maintained under continuous grazing and given pellets without fungal spores

^b^G-2: SSHs dewormed (1 mg Ivermectin/kg bw *pour on*), kept under rotational grazing and provided pellets without fungal spores

^c^G-3: SSHs dewormed (1 mg Ivermectin/kg bw *pour on*), kept under rotational grazing and given pellets containing 2 × 10^6^ spores of *Mucor circinelloides* + 2 × 10^6^ spores *Duddingtonia flagrans*/kg

The ERP was 6 weeks (1.5 months) in G-1 horses, 10 weeks (2.5 months) in G-2 horses and 16 weeks (4 months) in G-3 horses.

### Dynamics of strongyles egg-output

**Fig. 3:**
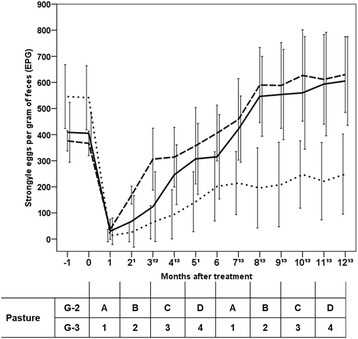
Kinetics of strongyle egg-output in Spanish Sport Horses under different pasturing regimes in an oceanic climate area (NW Spain). *Key*: (▬ ▬) G-1: continuous grazing and given pellets without fungal spores; (▬) G-2: four-paddock rotation system and pellets without fungal spores; (▪▪▪▪▪▪) G-3: four-paddock rotation system and pellets containing 2 × 10^6^ spores of *Mucor circinelloides* + 2 × 10^6^ spores *Duddingtonia flagrans*/kg. Points mean the average value, and error bars mean 2SD. Superscripts indicate time points differences between the strongyle egg-output values from G-1 and G-2 (**1**), G-2 and G-3 (**2**) and G-1 and G-3 (**3**)

The kinetics of strongyles eggs in feces are drawn in Fig. 3. In the horses kept under continuous grazing and given commercial pellets (G-1), the average EPG counts increased gradually from the 1st month after treatment (m. a. t.) to the end of the study, and average numbers from 306 (3rd m. a. t.) to 630 (12th m. a. t.) were achieved. FECR values between 80 and 0% were recorded until the end of the trial. One month after the deworming treatment, half of the horses passed eggs in their feces (PHR = 50%), and 15 days later (6 weeks after the deworming) all the equines were positive for the presence of strongyle eggs (Table 1).

The EPG numbers rose progressively by 1 m. a. t. in the G-2 horses (maintained under rotational grazing and provided commercial pellets), and values of approximately 300 EPG were observed at the 5th m. a. t. (Fig. 3). The egg-output levels increased above 500 EPG by 8 m. a. t., and the highest numbers were obtained at the end of the field trial (> 600 EPG). The average FECR values oscillated between 77 and 0%. Half of the horses were positive to the flotation test 6 weeks after treatment (PHR = 50%), and all were positive by 3 m. a. t. (14 weeks) (Table 1).

In the horses under rotational pasturing and supplemented with pellets containing fungal spores (G-3), the strongyle EPG counts rose steadily between the 1st and the 6th m. a. t., when an average value of 201 was observed. From this month to the 12th m. a. t., the egg-output numbers ranged between 196–249 EPG. By the 3rd m. a. t. (12 weeks) strongyle eggs were found in half of the SSHs, and all were positive for the presence of strongyle eggs at the 6th m. a. t. (26 weeks) (Table 1). The FECR percentages ranged between 83–54% throughout the study. PHR values greater than or equal to 50% were recorded until the 3rd m. a. t. (14 weeks). Only one horse in this group exceeded the threshold of 300 EPG, at the 9th m. a. t.

The data obtained in the first six months show that the average values of strongyle egg-output were 1.5-fold higher in G-1 than in G-2 and 3-fold in G-1 higher than in G-3. From the 7th to the 12th m. a. t., the average levels of EPG in G-1 and G-2 were very similar and were 2.6-fold higher than in G-3 (*χ*^2^ = 71.263, *df* = 2, *P* = 0.001).

Significant differences were observed in the values of strongyle egg-output according to the group of horses (*χ*^2^ = 59.138, *df* = 2, *P* = 0.001). By means of the Mann-Whitney U test, significant differences were identified between G-2 and G-3 (*U* = -5.988, *P* = 0.001) and between G-1 and G-2 (*U* = -5.650, *P* = 0.001) (Fig. 3).

### Adverse effects

None of the horses in G-3 rejected the pellets enriched with fungal spores. Normal appetite, digestive activity, reproductive behavior and breathing were recorded in all the horses. No signs of skin damage or disorder were observed during the assay. No alterations concerning pellet smell or thickness were observed, nor did hyphae grow on the pellet surface.

## Discussion

Grazing regimes offer horses nutrition and the opportunity to exercise and to interact with other individuals [23], but infection by gastrointestinal nematodes such as strongyles can be enhanced when infective stages (L3 larvae) are ingested with grass, and therefore, deworming is frequently needed. In the current study, the topical administration of ivermectin to horses under continuous pasturing provided successful results, based on the values achieved 14 days after treatment for the reduction in the fecal egg counts (FECR: 100%) and in the numbers of horses passing eggs of strongyles in feces (PHR: 100%), which agreed with the results of prior studies in the same area [7, 20]. The coprological assessment of anthelmintic efficacy comprises the estimation of FECR and thus concerns the effect on adult worms, but ivermectin presents a high activity against adult worms and larvae in the gut lumen, and low activity against encysted (hypobiotic) larvae [24]. Although the deworming was successful, the strongyle egg reappearance period was 6 weeks after treatment, probably due to the mobilization of encysted L3 larvae from the mucosa wall to the intestinal lumen to develop into adult stages [25]. Bearing in mind that in the current study, horses always fed on a pasture previously grazed by parasitized equines, another explanation involves the ingestion of L3s, which can attain the adult stage in 5–6 weeks, from the contaminated pasture [25]. The observation of EPG values higher than 300 (the threshold set at the beginning of the current study) from the 3rd month after treatment implies that deworming would be necessary again.

Several attempts have been made to avoid having horses that feed on grasslands become highly contaminated by L3 larvae, such as the regular collection of feces or pasture rotation [26]. In the present investigation, one group of horses maintained under grassland rotation involving a grazing period of 1.5 months and a rest phase of 4.5 months, was treated with ivermectin *pour-on*. Successful results were also observed according to the values of FECR (96%), although two out of eight horses continued to pass eggs in their feces (PHR: 75%). The strongyle egg reappearance period (ERP) was longer than in horses under continuous grazing (10 *vs* 6 weeks), as was the period in which all the equines became positive to the flotation test (14 *vs* 6 weeks). In addition to hypobiotic L3 larvae mobilizing to the gut lumen and developing to adult worms, the maintenance of horses in the same meadow for 1.5 months and then moving them to another paddock seemed to reduce their exposure to L3 strongyle larvae, and average egg-output values that were 49% lower than those in horses under continuous grazing during the first six months after treatment were obtained.

Rotational grazing is helpful to keep horses out of areas highly contaminated by L3 strongyles [27], and the numbers of infective L3s decrease with the length of the paddock resting period, so an infected pasture could return to a low level of risk of infection after a rest period of 3–6 months [28]. In the present investigation, horses re-entered the same pasture after a rest period of 4.5 months, and during this period (7–12 m. a. t.) the counts of the eggs of strongyles were 7% lower than in continuous grazing horses. These data appear to show that rotation decreases the level of pasture contamination, and consequently, infection is delayed or slowed down. However, the equines were re-infected, and at the end of the trial (12 m. a. t.), similar numbers of eggs of strongyles were recorded in the feces of either continuously grazing or rotationally grazing horses. Moreover, the observation of fecal egg-output values higher than 300 EPG starting at five months after treatment indicates that deworming is needed again after three pasture rotations. One possible solution to limit the numbers of strongyles in the pastures could rely on shortening the grazing period to less than 1.5 months [29] by increasing the number of rotated pastures. Because this is seldom possible, helpful measures appear necessary to reduce the viability of larvae of strongyles in the grass, or to avoid having the larvae develop from eggs inside the feces and move to the herbage.

The hatching of strongyle eggs and their further development from L1 to L3 larvae requires levels of soil moisture higher than 15–20% and takes 3 days at 25–35 °C, 15–24 days at 10 °C, or several weeks at temperatures < 10 °C [30]. Strongyle eggs can remain viable below 6 °C, although hatching does not occur [31]. In the area of study, average temperatures higher than 10 °C were recorded during all the months except between December and March (winter), when frost days were detected. The observation of high percentages of relative humidity (> 81%), together with notable rainfall values, except during the summer, and a negative water balance, only at the end of spring and at the beginning of summer, indicates that the soil moisture supports the growth of grasses nearly all year and offers a suitable environment for the development and survival of free-living stages of strongyles almost year-round. In addition to this, the eggs and larvae of strongyles are able to survive inside fecal pats and soil, even during cold periods [5]; under low levels of humidity and rainfall, L3s tend to remain in the feces, avoiding migration onto contiguous grass [5, 6], and fecal pats appear to become their main reservoirs [32]. Therefore, promising results could be attained by taking action against the strongyles in the feces.

Some saprophytic fungi are characterized by their antagonistic activity against different parasitic stages in the feces of infected animals. *Pochonia chlamydosporia* and *Mucor circinelloides* can destroy more than 50% of viable eggs of helminths such as *Calicophoron daubneyi*, *Toxocara canis* or *Trichuris* spp. [13, 18]. *Duddingtonia flagrans* is able to trap and then eliminate 94% of cyathostomin L3 when spores are directly sprayed on the feces of infected horses [33], and 27–99% when chlamydospores are given orally to horses from a similar climatic place (Curitiba, Brazil) [12]. Because the spores of these fungi can survive the industrial manufacturing of pelleted feed and the gastrointestinal tract, once administered to horses [16], in the present investigation, a third group of horses that was under rotational grazing was treated with ivermectin and provided twice a week with pellets industrially enriched with a blend of spores of *M. circinelloides* and *D. flagrans*. Deworming was effective (FECR: 99%), but one out of eight horses was positive to the flotation test 14 days after treatment (PHR: 88%). In comparison with horses under the same regime but feeding on pellets without spores, the ERP was extended by a further 1.5 months, and the period required for all the horses to shed strongyle eggs was extended by a further three months. Only one of the horses receiving pellets with spores exceeded the threshold of 300 EPG, and no adverse effects were recorded. Recently, an ERP of seven months has been reported among horses under continuous grazing and receiving daily pellets with spores of *M. circinelloides* and *D. flagrans*, and all the horses excreted eggs of strongyles by 15 months after their deworming [16].

When the pasture rotation ended (6 first months), a 50% reduction in the EPG values was observed in the horses given pellets with fungal spores compared with those given pellets without spores. After the horses re-entered the meadows they had previously grazed and completed the pasture rotation, the reduction in the EPG values was 59%. Since horses under rotational grazing were randomly distributed into two groups by the owner, as occurred with the designation of the pastures, it is suggested that the twice a week administration of pelleted feed with spores contributes to a lower risk of infection by strongyles. Previous investigations performed on continuously grazing horses showed that the numbers of fecal strongyle eggs decreased by 35–73% after providing them with a weekly ration of pellets with the mycelia of *D. flagrans* for six months [11], and the daily administration of pelleted feed with chlamydospores of *D. flagrans* was correlated with significant EPG reductions after 6 months (78%) and 12 months (67%) [24]. The results from the present research appear to indicate that fungi in the feces negatively influence the development of eggs and/or larvae in the feces, decreasing the level of contamination. Despite the high efficacy of *D. flagrans* in trapping and destroying strongyle larvae [11, 33], no data are available concerning the effect of *M. circinelloides* on strongyle eggs, although its ovicidal activity against the eggs of trematodes and ascarids has been demonstrated [15].

## Conclusions

The effective control of horse strongyles needs to include strategies to reduce the numbers of infective larvae in pastures, which will make it possible to decrease the frequency of anthelmintic treatments. In oceanic climate regions, rotational grazing contributes to delaying horse infection by strongyles, but the long-term persistence of the viable stages in the soil/herbage requires additional measures to avoid their migration from the feces to the grass, unless short grazing periods (< 6 weeks) or a high number of grazed pastures can be used. Supplementation twice weekly with pellets industrially manufactured with the spores of *M. circinelloides* and *D. flagrans* ensures the presence of these two parasiticidal fungi in the feces, which can reduce the development of strongyles in the environment and thus the risk of horse infection. The current results were obtained during a one-year period, and a more extensive follow-up study is in progress to gain information to confirm these initial findings.

## Abbreviations

FECR: Fecal egg-count reduction
PHR: Reduction of the numbers of horses shedding eggs
ERP: Egg reappearance period
EPG: Eggs per gram of feces
SSH: Spanish Sport Horses
L1: First-stage larva
L2: Second-stage larva
L3: Third-stage larva
m. a. t: Month after treatment
